# Towards a harmonised framework for developing quality of care indicators for global health: a scoping review of existing conceptual and methodological practices

**DOI:** 10.1136/bmjhci-2021-100469

**Published:** 2022-01-25

**Authors:** Lilian Dudley, Puni Mamdoo, Selvan Naidoo, Moise Muzigaba

**Affiliations:** 1 Department of Global Health, Faculty of Medicine and Health Sciences, Stellenbosch University, Stellenbosch, Western Cape, South Africa; 2 Department of Maternal, Newborn, Child and Adolescent Health and Ageing, World Health Organization, Geneva, Switzerland

**Keywords:** common data elements, health information systems, outcome and process assessment, healthcare, outcome assessment, health care, patient care

## Abstract

**Objectives:**

Despite significant advances in the science of quality of care measurement over the last decade, approaches to developing quality of care indicators for global health priorities are not clearly defined. We conducted a scoping review of concepts and methods used to develop quality of healthcare indicators to better inform ongoing efforts towards a more harmonised approach to quality of care indicator development in global health.

**Methods:**

We conducted a systematic search of electronic databases, grey literature and references for articles on developing quality of care indicators for routine monitoring in all healthcare settings and populations, published in English between 2010 and 2020. We used well-established methods for article screening and selection, data extraction and management. Results were summarised using a descriptive analysis and a narrative synthesis.

**Results:**

The 221 selected articles were largely from high-income settings (89%), particularly the USA (46%), Canada (9%), UK (9%) and Europe (17%). Quality of care indicators were developed mainly for healthcare providers (56%), for benchmarking or quality assurance (37%) and quality improvement (29%), in hospitals (32%) and primary care (26%), across many diseases. The terms ‘quality indicator’ and ‘quality measure’ were the most frequently encountered terms (50% and 21%, respectively). Systematic approaches for quality of care indicator development emerged within national quality of care systems or through cross-country collaborations in high-income settings. Maternal, neonatal and child health (33%), mental health (26%) and primary care (57%) studies applied most components of systematic approaches, but not consistently or rigorously.

**Discussion:**

The current evidence shows variations in concepts and approaches to developing quality of care indicators, with development and application mainly in high-income countries.

**Conclusion:**

Additional efforts are needed to propose ‘best-practice’ conceptual frameworks and methods for developing quality of care indicators to improve their utility in global health measurement.

## Introduction

In 2015, the WHO prioritised improving quality of care (QoC) for women, new-borns and children and conceived a vision in which ‘Every woman, new-born, child and adolescent receives quality health services throughout the continuum of their life course and level of care’.^1^ The WHO published frameworks and standards for improving the QoC for Maternal and Newborn Health (MNH) in health facilities and for children and young adolescents, defining eight domains for assessment, improvement and monitoring the provision and experience of care.^2 3^ The WHO also led the development of Maternal, Neonatal and Child Health (MNCH) QoC indicators for a global network for improving QoC for MNCH.^4^

Several MNH measurement initiatives emerging from the Millennium Development Goals and the Sustainable Development Goals have generated a substantial volume of indicators, many of which partially overlapped and lacked adequate documentation or guidance.^5^ Many MNH QoC metrics were not based on clear concepts of causality and were insufficiently supported by research evidence.^6^ Of more than 1400 indicators of the MNH care continuum, only 6.7% met all requirements for scientific soundness by providing a full description and empirically testing validity, reliability and feasibility.^7^ MNH standards and indicators of coverage and quality performed poorly in validity, feasibility and usability assessments in low/middle-income countries (LMICs).^6–8^

Three major reports on the global quality of healthcare agenda highlighted the need for a health systems perspective and better QoC measurement in MNCH QoC programming.^9–11^ These reports, however, did not review or recommend methods for improving the development of measures of quality of healthcare in global health, albeit acknowledging the existing gaps in this area. The release of these reports and the MNCH QoC standards sparked dialogue on how best to measure MNCH QoC, particularly focusing on appropriate methods for developing QoC metrics for routine and/or periodic monitoring and reporting in LMICs.^9^ We chose to conduct a scoping review as the most appropriate research method to systematically map the available literature, to identify key concepts, theories, sources of evidence and gaps in the research on the development of QoC measures for global health.^12^

This scoping review sought to describe and critically examine the literature between 2010 and 2020 on: (1) the terminology and concepts used in QoC metrics; (2) the purposes for the development of and use of QoC indicators and (3) methods used in QoC indicator development; with a view to better inform QoC indicator development in global health.

## Methods

In this article, QoC is defined as ‘the extent to which healthcare services provid desired health outcomes to individuals and patient populations. To achieve this, the care must be safe, effective, timely, efficient, equitable and people centred’.^13 14^

This scoping review was guided by the adapted framework of Arksey and O’Malley and sought to capture the variability of QoC indicator development methods and concepts across diverse clinical and healthcare contexts.^15–17^ The study protocol was developed in consultation with WHO experts and was guided by the Preferred Reporting Items for Systematic Reviews and Meta-Analyses—Extension for Scoping Reviews Checklist.^18^ The protocol was registered on the Open Science Forum (https://osf.io/) in December 2020.

### Search strategy

We searched PubMed, CINAHL/EBSCOhost, Embase, the Cochrane Library and Health Systems Evidence databases, using a search strategy based on MeSH terms (online supplemental file). We also searched the grey literature and reference lists of primary articles identified. The last search was on 11 January 2021.

10.1136/bmjhci-2021-100469.supp1 Supplementary data

### Eligibility criteria

We included publications which described or applied methods to develop QoC indicators for routine monitoring in all healthcare settings and populations. All study designs, reviews, reports, book chapters and conference presentations published in English between 2010 and 2020 were included. We excluded studies of patient reported outcome measures (PROMs), patient reported experience measures (PREMs), quality of life, economic evaluations, evaluations of quality improvement interventions and publications that lacked sufficient rigour. ‘Seminal’ publications on developing QoC metrics prior to 2010 were included.

### Study selection and data extraction

The search results were loaded into Covidence for the screening of titles, abstracts, full texts and for data extraction.^19^ Two researchers reviewed all titles, abstracts and full texts independently, and included articles if there was agreement. A third researcher acted as arbitrator to resolve conflicts. We piloted a data extraction form, and data extraction was double-checked by a second reviewer before uploading the data. A rigorous data quality and verification process were conducted in Microsoft Excel V.16.^20^ Quantitative data were coded and exported into STATA V.14 for analyses.^21^

### Data analysis and reporting

We described the distribution of included studies by publication year, country, income level, healthcare setting, population or programme, study design, purpose (use and users) and methods or approaches to the development and assessment of QoC metrics. Qualitative data were constructed into themes for content analysis to provide an intuitive, perceptible view of the body of knowledge. Articles on developing QoC indicators for MNCH, mental health and primary care were analysed in greater depth. We assessed the rigour of this subset of studies, adapting criteria for QoC metrics development from several sources.^18 22–24^

## Results

### Characteristics of included articles

**Figure 1:**
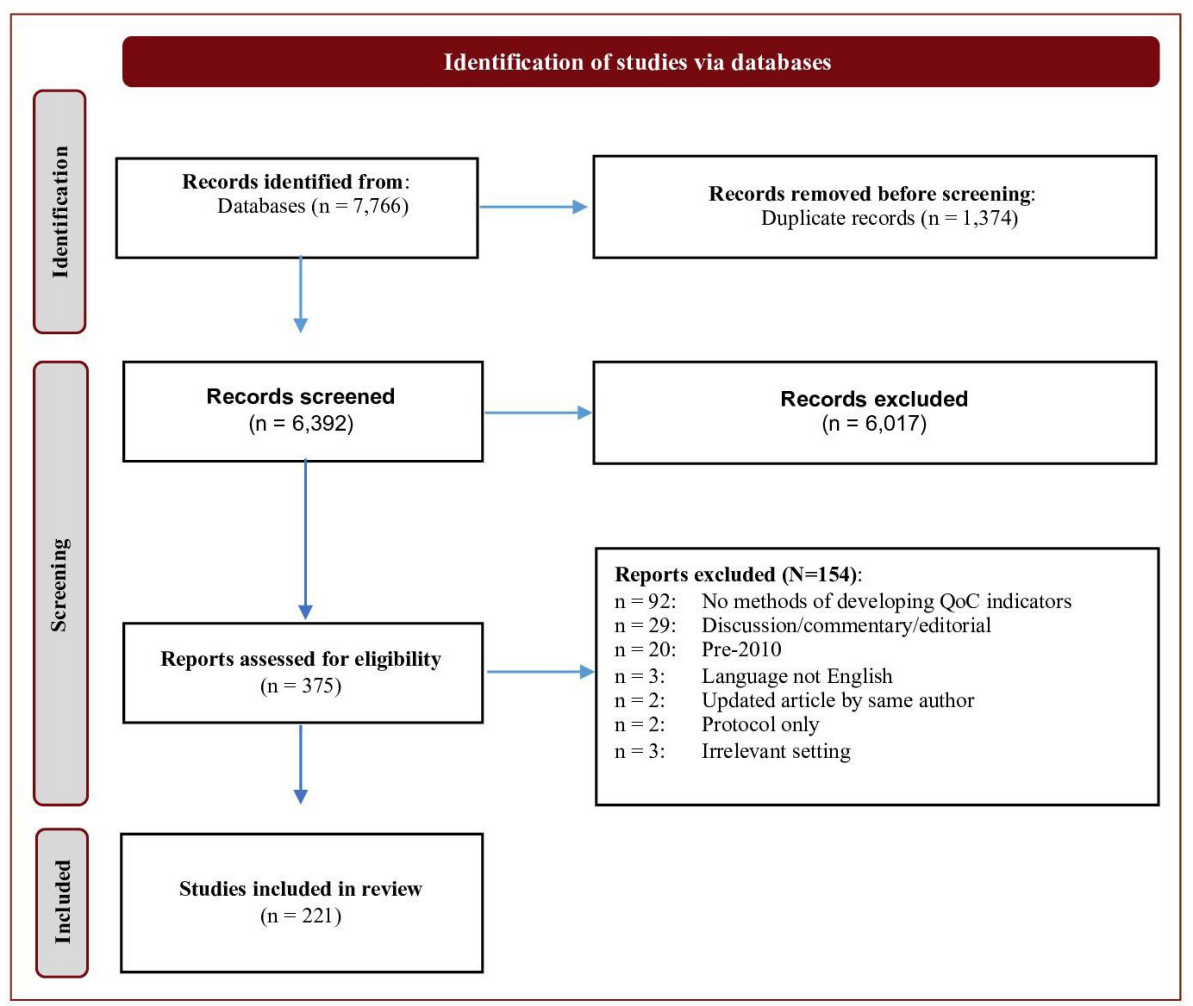
PRISMA diagram of study selection and screening. PRISMA, Preferred Reporting Items for Systematic Reviews and Meta-Analyses; QoC, quality of care.

**Figure 2:**
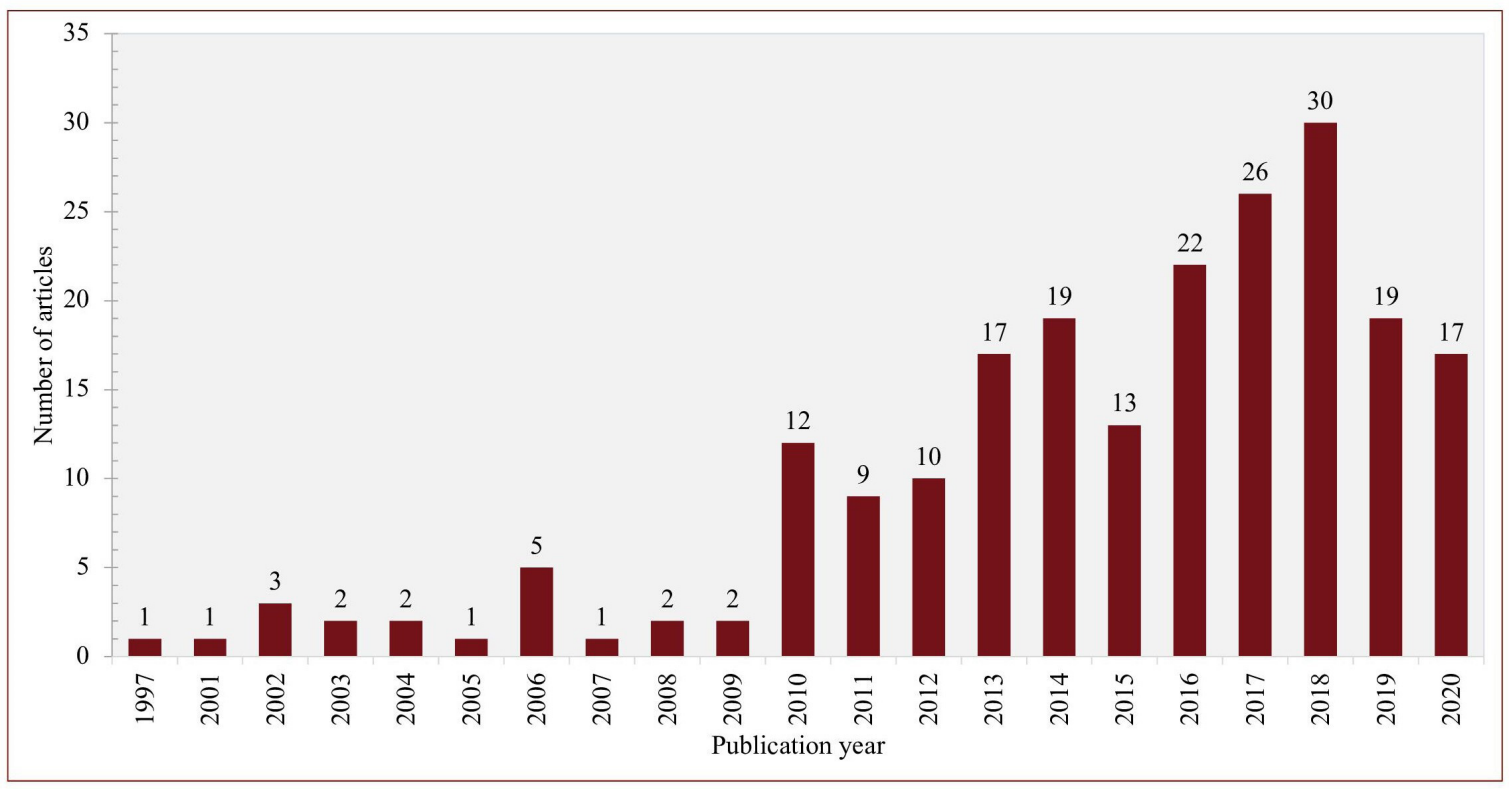
Distribution of articles by publication year (2010–2020).

The literature search identified 7766 references, of which 1374 were duplications. We excluded 6017 after screening the titles and abstracts, and a further 154 after full text screening, resulting in a final dataset of 221 articles (figure 1). Of these, 201 were published between 2010 and 2020 with a slight increase in the number of publications per year over time (figure 2), and 20 were seminal papers published before 2010. Publications from high-income settings (178; 89%) predominated, with only 23 (11%) from LMICs. Most studies were from a single country (156; 78%), particularly the USA (93; 46%), Canada (18; 9%) and the UK/Ireland (18; 9%). The 37 (17%) studies from Europe were distributed across several countries. There were 35 multicountry studies, mainly from Europe and the Organisation of Economic Development (OECD). The LMIC studies were from Asia (8; 4%), Africa (6; 3%), Latin America (3; 1%), and four were reviews of indicators for low resource settings.

The studies were grouped into (1) Publications which described concepts or methods of developing QoC indicators (51; 23%), and (2) studies in which new QoC indicators were developed (170; 77%). We synthesised concepts and methods for developing QoC indicators from the first group, which provided a framework for analysis of the second group of studies.

### Terminology

**Figure 3:**
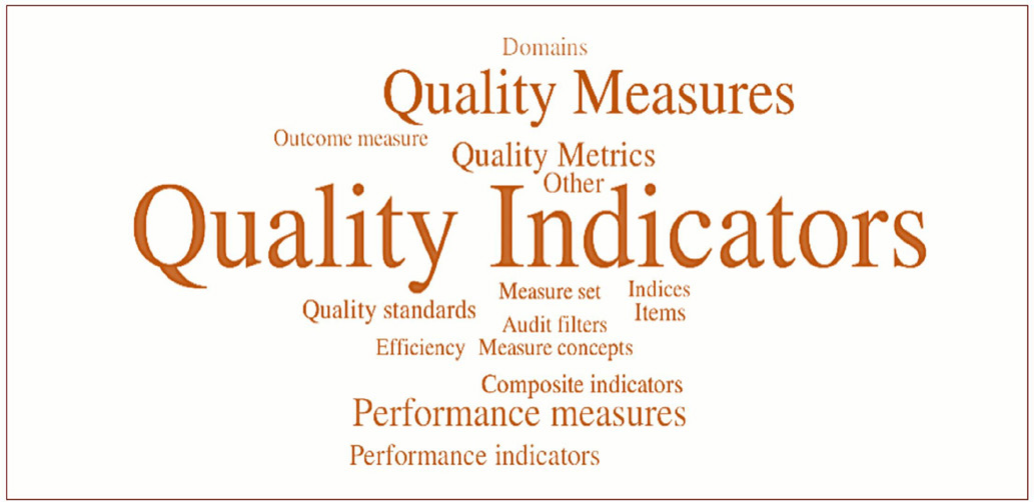
WordCloud of terms used in QoC measurement reflecting frequency of use in included articles. QoC, quality of care.

The terms ‘quality indicator’ (50%), ‘quality measure’ (21%), ‘performance measure’ (8%), ‘quality metric’ (6%) and ‘performance indicator’ (3%) were used as conceptual terms for QoC measurement. The term ‘quality measures’ predominated in USA publications, whereas ‘quality indicators’ was most frequent elsewhere. The terms were occasionally used interchangeably, with overlapping definitions of ‘quantitative, systematic, evidence based methods of measuring aspects of healthcare’, applied to ‘structures, processes and outcomes’ of care, and compared against a ‘standard’ of care.^25–30^ Figure 3 provides a WordCloud of these terms as identified in the literature.

### Characteristics of included studies (pooled analysis)

In the 221 studies, healthcare settings were mainly hospitals (71; 32%) followed by primary care facilities (58; 26%), and 22% of the studies were relevant across settings. The main topics were chronic diseases (26; 12%), mental health (26; 12%), child health (20; 9%), maternal health (13; 6%), surgery (13; 6%), cancer (12; 5%) and trauma care (9; 4%). Only six studies addressed infectious diseases, of which three focused on HIV care, two on antibiotic prescribing and one on tuberculosis control. The main purposes of developing QoC indicators were for benchmarking or quality assurance (81; 37%), quality improvement (64; 29%), testing and validation of the indicators (27; 12%), contracting or reimbursement (14; 6%) and local or international comparisons (14; 6%). Most studies indicated that the QoC indicators served multiple purposes. Targeted users were healthcare providers (124; 56%), researchers or measure developers (20; 9%), healthcare funders or payers (17; 8%), professional groups or organisations (7; 3%), policy makers (7; 3%) and regulatory or accreditation bodies (5; 2%). Most indicated relevance for multiple users.

### Methods of developing QoC indicators

**Figure 4:**
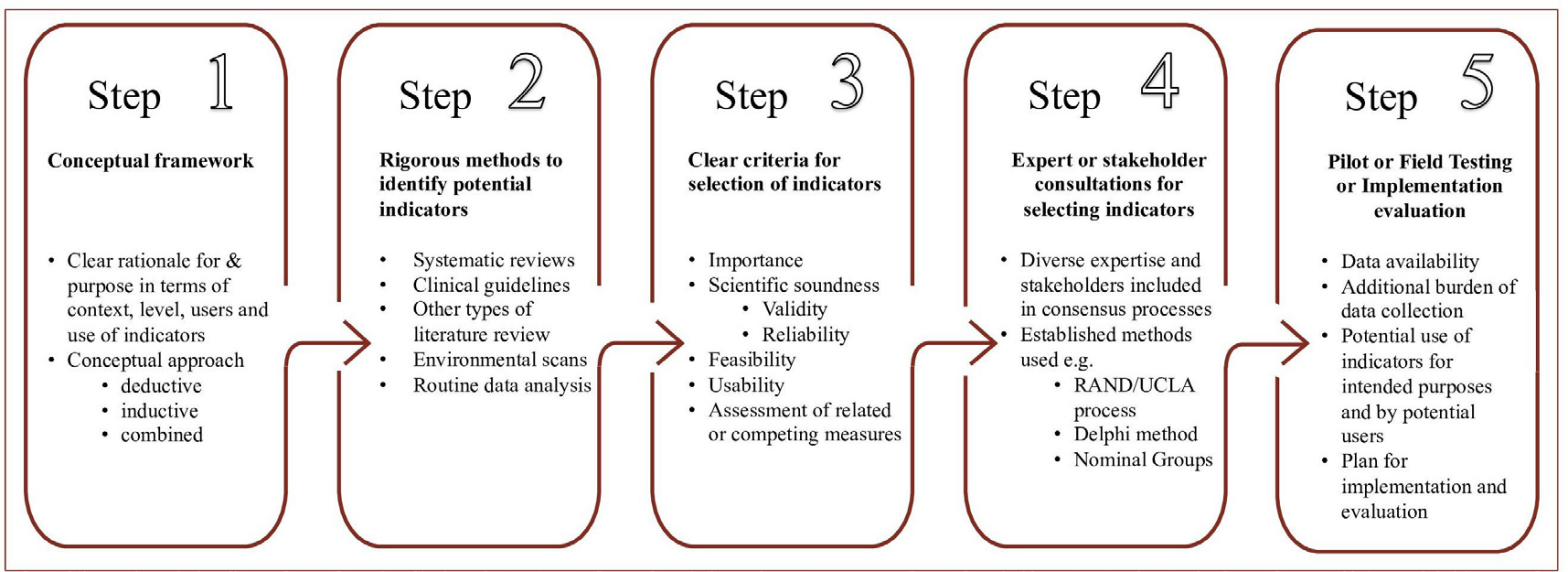
Core steps to guide the development of quality of care indicators. RAND, Research ANd Development; UCLA, University of California Los Angeles.

The methods of developing QoC indicators were refined for local purposes in the USA, the UK and several OECD countries through key institutions including the Institute of Medicine, the Agency for Healthcare Research and Quality (AHRQ), National Quality Forum (NQF), the RAND Corporation, The National Institute for Health and Care Excelence (NICE), the National Health Service (NHS) and numerous academic and research institutions.^31 32^ The OECD Healthcare Quality Indicators (HCQI) project, implemented across over 30 high and upper middle income countries, adapted approaches for cross-country comparisons.^33^ We distilled five core steps recommended in methods papers as a guide to the development QoC indicators.^22–24 32 34 35^ These steps are summarised in figure 4.

#### Step 1: conceptual framework

Conceptual frameworks clarify the purpose and focus for QoC indicators by defining what concepts or dimensions of quality of healthcare should be measured and how, in principle, they should be measured. Indicator frameworks for specific diseases have been developed using concept mapping with extensive stakeholder engagement.^32^ Quality indicators have also been integrated into in broader health systems performance frameworks within countries or by international organisations for cross-country comparisons.^36 37^

Conceptual approaches to developing QoC indicators were either ‘deductive’ (from concept to data) or ‘inductive’ (from data to concepts).^24^ Deductive approaches, also described as ‘systematic’, start with an important QoC concept, systematically draw on scientific evidence to identify potential indicators and use an expert consensus process to select final indicators. Inductive approaches which are less frequently applied, start with data, either arising from real life critical incidents or by evaluating and querying data for variation to inform the QoC concept.^24 34^

#### Step 2: identifying potential indicators from evidence or clinical guidelines

The best quality evidence for QoC indicators is from well-designed randomised controlled trials, meta-analysis or systematic reviews.^29 38^ The NQF further requires that the relationships of the health outcome to processes or structures of care should be supported by a systematic assessment and grading of the quality and consistency of the evidence.^22^ The selection of QoC indicators from clinical guidelines, similarly, should be informed by the methodological quality of the guideline (usually assessed by a critical appraisal).^35 39^ The Guidelines International Network-McMaster Guideline checklist is used for assessing guidelines and their performance measures.^35 40 41^

#### Step 3: criteria for the selection of indicators

The NQF criteria for the assessment and selection of QoC indicators include importance of the indicators, their scientific acceptability (based on validity and reliability), usability, feasibility of measurement and whether there are any competing indicators.^22 42^ The AHRQ criteria include the size of impact on patient health, value for money, feasibility and measurability of the indicator at issue. These sets overlap with NICE’s criteria to appraise the validity of indicators within six domains of ‘importance, evidence base, specification, feasibility, acceptability and risk’.^23^ Other proposed criteria included reproducibility, sensitivity to change and actionability.^32 43^

Definitions of common selection criteria are provided in the online supplemental file. On the other hand, there is a number of indicator validity testing approaches which were drawn from the literature. Content validity assesses whether the content of an indicator is consistent with professional knowledge and the outcomes of high-quality care.^14^ Face validity supports links between structure, processes and/or outcomes in the healthcare system and is usually informed by expert consensus.^29 44^ Construct validity assesses if the indicator relates to other indicators in a manner consistent with a theoretically derived hypothesis.^24^ Criterion validity is the extent to which one purported indicator of quality is correlated with other, better ‘gold standard’ indicators of the same phenomenon.^45^ Content and face validity are minimal requirements for validity assessment, but it is preferable that construct and criterion validity also be assessed.^45^

#### Step 4: expert consensus and stakeholder consultations

Consensus methods, such as the Delphi process and Nominal group methods, address gaps or inconsistencies in the evidence, and synthesise accumulated expert opinion in selecting final indicators.^46 47^ The “ Research and Development/ University of California Los Angeless “ (RAND/UCLA) appropriateness method includes a modified Delphi process with a panel of experts who consider the evidence and rate the ‘appropriateness’ of each indicator.^48^ The selection, composition and functioning of such panels are important considerations especially when the available evidence for the development of indicators is limited or contradictory, or the results need to be extrapolated to different populations or settings.^24^

#### Step 5: field testing and implementation

Field testing involves field or pilot studies in settings where the QoC indicators are to be used, preferably with the involvement of the intended users, to determine the availability of data for the QoC indicators, any potential burden for data collection, and the utility of indicators in relation to the intended use.

### Characteristics of included studies (sub-analysis)

We used the above ‘best practice’ methods to guide a sub-analysis of MNCH studies (n=33), mental healthcare (n=26) and primary care (n=57) as reported in table 1. These studies were also mainly from high-income countries (HICs) except that more MNCH studies (21%) were conducted in, or for, LMICs. Similar proportions of the MNCH and mental health studies were in hospitals (42% and 39%, respectively), but relatively few MNCH studies (12%) were in primary care settings compared with mental health (46%), and relatively more MNCH were across settings (33% and 12%).

**Table 1:** Description of MNCH, mental health and primary care studies

Categories		Variables	MNCH, n=33		Mental health, n=26		Primary care, n=57	
			n	%	n	%	n	%
World Bank classification of country		HIC	24	72.7	25	96.2	49	86.0
		HIC and MIC	0	0	1	3.8	0	0.0
		LMIC	7	21.2	0	0	6	10.5
		Not reported	2	6.1	0	0	2	3.5
Study setting		Hospital	14	42.4	10	38.5	0	0.0
		Primary care	4	12.1	12	46.2	57	100.0
		Transition care	2	6.1	1	3.8	0	0.0
		All	11	33.3	3	11.5	0	0.0
Purpose of developing QoC metrics	Users	Providers	24	72.7	14	53.8	33	57.9
		All	4	12.1	2	7.7	3	5.3
		HMO’s, managed healthcare groups	0	0	1	3.8	4	7.0
		Professional society	0	0	4	15.4	1	1.8
		Policy makers	0	0	0	0	3	5.3
		Other/NR	2	6.1	5	19.2	7	12.3
		Funders	3	9.1	2	7.7	6	10.5
	Uses	Quality improvement	12	36.4	8	30.8	18	31.6
		Benchmarking or QA	12	36.4	8	30.8	19	33.3
		Testing and validation	3	9.1	3	11.5	7	12.3
		Contracting or reimbursement/VBC	2	6.1	0	0	4	7.0
		Local or international comparisons	2	6.1	4	15.4	4	7.0
		Other	2	6.1	3	11.5	5	8.8
Clear rationale for need for metrics		Yes	30	90.9	25	96.2	53	93.0
		N/A	3	9.1	1	3.8	4	7.0
Main term used for QoC metrics		Quality indicator	17	51.5	11	42.3	33	57.9
		Quality measure	11	33.3	8	30.8	5	8.8
		Performance measures	2	6.1	3	11.5	3	5.3
		Performance indicators	0	0	1	3.8	2	3.5
		Other	3	9.1	3	11.5	11	19.3

HIC, high-income country; HMO, Health Maintenance Organization; LMIC, low/middle-income country; MIC, middle income country; MNCH, maternal neonatal and child health; N/A, not applicable; NR, not reported; QA, quality assurance; QoC, quality of care; VBC, value based contracting.

As shown in table 1, the main purposes of QoC indicator development were for quality improvement (31%–36%), benchmarking or quality assurance (31%–36%), testing and validation of metrics (9%–12%) and local or international comparisons (6%–15%). The users varied, with healthcare providers targeted most in MNCH (73%), professional societies prominent in mental health (15%) and funders less represented across the groups.

As shown in table 2, most studies (>80%) performed well against the four initial steps involved in indicator development but very few (19%) conducted field-testing of the indicators (fifth step). Although few studies reported using a conceptual framework, most provided a clear rationale for the need for indicators (98%). Most approaches to developing QoC metrics were deductive (36%) or combined deductive and inductive approaches (49%). The methods used to identify potential QoC metrics generally included a review (81%), but these ranged from literature reviews or environmental scans where the methods were not clearly described to more rigorous well described systematic reviews. Approaches varied across the three programmatic areas, with mental health using the most rigorous methods, and combining methods (literature review, clinical guidelines, existing indicators and/or routine data) extensively. MNCH and primary care studies used reviews only or combined methods, and a few used routine data. Only primary care studies (13%) used existing indicators. Eleven (16%) of 70 studies that conducted reviews, described the indicators or compiled a list of potential indicators, but did no further assessments of the indicators. Just over 80% of the studies in the three groups provided clear criteria for the assessment and selection of QoC indicators. They mostly included validity (61%) and feasibility (59%) assessments, but fewer assessed reliability (43%) and usability (39%) of the indicators. Validity testing focused mainly on face and content validity, with very few studies reporting construct or criterion validity of metrics. Comparisons of the new indicators to related or competing indicators were rarely conducted (12%). Panel or group consensus methods were used in most studies (88%), with the RAND/UCLA (26%) and Delphi (23%) methods most commonly used. Mental health studies involved mainly experts, and MNCH and primary care studies engaged a mix of experts, healthcare providers and multiple stakeholders. About one-third used a combination of methods (panel rankings, statistical analysis, pilot studies), and some relied solely on expert panel rankings, with a few reporting other methods to assess and select indicators. Very few studies described piloting or field testing of the selected QoC indicators in the settings for which they were intended (19%) or monitored implementation of the indicators.

**Table 2:** Performance of MNCH (n=33), mental health (n=26) and primary care (n=57) studies against key steps for developing quality of care indicators

	1. Conceptual approach and framework	2. Methods used for identifying potential metrics	3. Clear criteria for selection of indicators	4a. Consultation or consensus methods used	4b. Which stakeholders were consulted	5. Were indicators field tested or piloted
Overall	Clear rationale (98%)	Literature or clinical guidelines reviewed (81%)	Clear criteria for assessment and selection (81%)	Panel or group methods used (88%)		Pilot or field testing done (19%)
Specific method or approach used	Deductive (36%)	Systematic reviews (20%)	Validity (61%)	RAND/UCLA (26%)	Experts (45%)	
	Inductive (4%)	Rapid reviews, overviews or scoping reviews (13%)	Reliability (43%)	Delphi (23%)	Healthcare providers (18%)	
	Combined (49%)	General literature review (24%)	Feasibility (59%)	Nominal group (9%)	Multiple stakeholders (22%)	
		Clinical guidelines review (10%)	Usability (39%)	Combination (12%)	Not reported (14%)	
		Environmental scan (15%)	Related and competing measures (12%)	Other for example, workshop, focus groups, interviews (21%)		
		Routine data (4%)				

MNCH, Maternal Neonatal and Child Health; RAND/UCLA, Research and Development/ University of California Los Angeless.

## Discussion

We characterised the QoC measurement landscape for the period 2010–2020, in terms of the rationale, key concepts and methods used to develop QoC indicators. Terms for QoC metrics tended to be consistent within countries, with ‘quality measures’ used almost exclusively in USA and ‘quality indicators’ used elsewhere. Many studies did not define their terms, or used terms interchangeably. Wider agreement on common concepts and terms is needed in developing QoC indicators for global purposes.^32^

The QoC landscape was strongly influenced by the USA, the UK and OECD countries, with a focus on diseases prevalent in these countries. The few publications from LMICs focused mainly on local testing of global MNH indicators. The leading HICs had national policy frameworks, and well-functioning systems for the assessment of the QoC for accreditation, reimbursement, public disclosure and quality improvement.^31 32^ Healthcare providers were important users of QoC indicators. Most studies provided a clear rationale for the need for QoC metrics and applied deductive, systematic approaches using various types of evidence to select potential indicators, and assessed these against clear criteria using expert consensus to select the final indicators.^22 34 35^ Assessments of indicators were, however, not consistently or rigorously applied, field testing of indicators for usability in practice was not routinely undertaken, and comparisons to existing indicators were infrequent.

Despite extensive experience and rigour in the development of paediatric QoC indicators for use at micro and meso levels in HICs for quality improvement and accountability, there was limited translation of these practices to the development of global MNCH metrics.^32 49 50^ The rapid proliferation of global MNH indicators, with definitional variations, few shared indicators, low scientific soundness, focus on facility inputs and processes rather than outcomes or impacts, and limited feasibility and usability in LMICs has been widely reported.^5–8 51 52^ A review provided guidance on assessing the validity of MNH indicators, but no guidance was found on reliability, feasibility and usability assessment in different settings.^45^

In the field of mental health, pressure from healthcare and payment reforms led to development of a large number of quality indicators. Less than 10% of more than 500 behavioural health measures in the USA were used in major quality reporting programmes or were endorsed by the NQF.^53^ Possible reasons included extensive duplication, measures originating from research which might not be generalisable or practical, and many measures had insufficient evidence to support their usefulness.^54^ Cross-country reviews of mental health QoC also found wide variation in the scope, intended use and degree of development, with measures cutting across a broad range of domains.^55 56^ Many countries also lacked adequate quality measurement infrastructure for cross-country comparisons.^54 55^

This highlights some of the challenges to identifying a set of common indicators for global QoC, including varying expert opinions and interpretation of the evidence within different settings.^31^ Collaborations through the OECD or EU to develop QoC metrics sought to address some of the challenges by promoting consistency in a few elements across national boundaries.^57^ They were guided by frameworks and principles including that ‘the common set of measures is a small number of key measures that are useful for both choice and improvement, rather than a comprehensive set of all acceptable measures’.^31 43 57^ The HCQI recognised that a common set of measures should be parsimonious and not impose undue burdens on those who provide data; and should help providers improve the delivery of care and help all stakeholders make more-informed decisions.^57^

Other important considerations identified were the types of indicators, their purpose and the criteria used for selection of global indicators.^14^ With respect to the types of indicators, the balance of structure, process and outcome indicators for international comparisons may differ from local or national choices.^14 58^ Process indicators are useful for ‘formative’ purposes to inform local quality improvement strategies, as they are more sensitive to changes, are able to detect deficits in care more rapidly and capture aspects of care which are more important to patients. The development of QoC indicators for ‘summative’ purposes depends more on output and outcome indicators. These present different challenges in terms of data sources and require a higher level of precision as the judgements have implications for licensing, public accountability and remuneration.^14^

For cross-country comparisons, outcome and impact measures represent the endpoints of QoC at a macro level by assessing whether the health system is achieving the desired goals and meeting the patients’ needs.^43 58^ Thus, despite some limitations, outcome measures may be preferred in international benchmarking and global comparisons of QoC. The importance of impact, outcome and output metrics at a global level has been highlighted, rather than the many overlapping input and process indicators used for monitoring implementation of vertical programmes.^10^

Careful consideration is also needed for criteria for scientific appropriateness of global indicators. Direct comparisons of care should only be made when guidelines and criteria are similar, requiring a strong international evidence base for such guidelines and indicators.^38 40 41^ The importance of an integrated approach to the development of quality indicators for quality assurance systems and clinical guidelines has recently been highlighted.^35 59^ Criteria, such as feasibility, may be more important as international comparisons depend on existing data and information systems which may present serious limitations in many countries. The choice of quality indicators also depends on the needs of stakeholders, with criteria such as ‘meaningfulness’ proposed for comparisons across providers, regions and/or countries.^14^

There is a need to differentiate criteria to select global indicators based on the intended purpose of the indicators in terms of use, users and the level of use in health systems.^58^ This further highlights the importance of clarifying the purpose of global QoC indicators, and the need for greater harmonisation of approaches to ensure methodological, contextual and managerial fitness for purpose at country and global levels.^58^

### Limitations

The measurement of QoC is a broad and complex field including many dimensions, perspectives, approaches and settings. Our review focused on methods of developing routine, largely quantitative QoC indicators in healthcare settings. We did not review methods of developing PREMs or PROMs, economic evaluations, nor of measures to evaluate quality improvement interventions. The language and time period restrictions may have excluded other relevant studies. The included studies were mainly from HICs, indicating a substantial gap in evidence on the development of QoC indicators in LMICs or for global purposes.

### Implications for practice, policy and research

Adequate national QoC and data infrastructure systems, with supportive cross-country collaborations, are fundamental to the development and use of global QoC indicators. The Lancet Commission advocated the development of ‘country-led’ QoC measurements systems, and the strengthening of national health information systems to facilitate the measurement of QoC.^10^ National QoC indicator development efforts should be guided by the principles of evidence best practices in QoC measurement, alignment with national priorities and scientific soundness to ensure indicators are feasible to measure, usable and meaningful in settings where they are to be applied. On a global scale, where common measurement and monitoring of QoC efforts across countries would be needed for accountability and policy, there is a need for innovative research to develop a small set of indicators that carry most of the relevant information, thereby alleviating the need to measure every aspect of care. Evidence from this research should form the basis for developing standardised guidelines on how to develop global QoC indicators to guide future efforts around QoC indicator development across disease areas.

## Conclusion

Our review shows variations in concepts and approaches used to develop QoC indicators, with development and application of such indicators being mainly in HICs. Additional research efforts are needed to propose ‘best-practice’ conceptual frameworks and methods for developing QoC indicators to improve their utility in global health measurement.

## Data Availability

Data are available in a public, open access repository. Information retrieved through literature review is freely available by accessing the search strategy provided in the supplementary information.
