# Supplementary material for: Towards a harmonised framework for developing quality of care indicators for global health: a scoping review of existing conceptual and methodological practices
Source: BMJ Health Care Inform. 2022 Jan 25;29(1):e100469. doi: 10.1136/bmjhci-2021-100469 (PMC8796246; doi:10.1136/bmjhci-2021-100469)
Supplement: Supplementary data [file bmjhci-2021-100469supp001.pdf]

SUPPLEMENTARY FILE

1. Search strategy

Database: Ovid MEDLINE(R) and In-Process & Other Non-Indexed Citations <1946 to January 06, 2021>
Search Strategy:
----
1 *"Quality of Health Care"/
2 *Quality Assurance, Health Care/
3 *Quality Improvement/ or *Total Quality Management/
4 ((health care or healthcare) adj2 (quality or assurance or improvement)).tw.
5 1 or 2 or 3 or 4
6 Benchmarking/
7 metric*.tw.
8 Reference Standards.mp.
9 quality indicator*.tw.
10 (Outcome and process assessment).mp.
11 measurement*.tw.
12 Health Services Research/cl, mt, og, st, td
13 *Quality Indicators, Health Care/
14 *Health Status Indicators/
15 6 or 7 or 8 or 9 or 10 or 11 or 12 or 13 or 14
16 5 and 15
17 maternal health services.mp.
18 Reproductive Health services.mp.
19 child health services.mp.
20 (tuberculosis or HIV*).tw.
21 Communicable Diseases/
22 Chronic Disease/
23 Noncommunicable Diseases/
24 Mental Health Services/
25 hospital care.mp.
26 clinical care.mp.
27 surgical care.mp.
28 Primary Health Care/
29 Nursing Care/
30 17 or 18 or 19 or 20 or 21 or 22 or 23 or 24 or 25 or 26 or 27 or 28 or 29
31 16 and 30
32 limit 31 to (english language and humans and yr="2000 -Current")

Database: Embase <1996 to 2020 Week 53>
Search Strategy:
----
1 *health care quality/
2 health care quality assurance.mp.
3 Quality Improvement.tw. or *Total Quality Management/
4 ((health care or healthcare) adj2 (quality or assurance or improvement)).tw.
5 1 or 2 or 3 or 4
6 Benchmarking/
7 metric*.tw.

8 Reference Standards.mp.
9 quality indicator*.tw.
10 (Outcome and process assessment).mp.
11 measurement*.tw.
12 *Health Status Indicator/
13 6 or 7 or 8 or 9 or 10 or 11 or 12
14 maternal health services.mp. or maternal health service/
15 Reproductive Health services.mp.
16 child health services.mp. or child health care/
17 (tuberculosis or HIV*).tw.
18 communicable disease/
19 Chronic Disease/
20 non communicable disease/
21 mental health service/
22 hospital care.mp.
23 clinical care.mp.
24 surgical care.mp.
25 Primary Health Care/
26 Nursing Care/
27 5 and 13
28 14 or 15 or 16 or 17 or 18 or 19 or 20 or 21 or 22 or 23 or 24 or 25 or 26
29 27 and 28
30 limit 29 to (human and english language and yr="2000 -Current")

Cinahl EBSCOhost
Published Date: 20000101-20211231
Narrow by Language: - english

#	Query
S16	S8 AND S12
S15	S8 AND S12
S14	S8 AND S12
S13	S8 AND S12
S12	S9 OR S10 OR S11
S11	TX mental health services OR TX (hospital care or inpatient care) OR TX surgical care OR MH primary health care OR TX nursing care
S10	TX communicable diseases OR TX (chronic disease or chronic illness or long term conditions or chronic conditions) OR MH noncommunicable diseases [mesh]
S9	TX maternal health services OR MH reproductive health services [mesh] OR TX child health services OR TX ((tuberculosis or HIV))
S8	S3 AND S7
S7	S4 OR S5 OR S6
S6	MH quality indicators OR MJ health status indicators
S5	TX quality indicator* OR TX (Outcome and process assessment) OR TX (measurement tool or assessment tool)
S4	MH benchmarking OR TX metrics OR TX reference standards
S3	S1 OR S2

S2	MH quality of health care OR MH quality assurance in healthcare OR MH quality improvement
S1	AB ((health care or healthcare) N2 (quality or assurance or improvement))

Cochrane Library issue 1 of 12, January 2021

#113 MeSH descriptor: [Quality of Health Care] this term only
#114 MeSH descriptor: [Quality Assurance, Health Care] this term only
#115 MeSH descriptor: [Quality Improvement] this term only
#116 MeSH descriptor: [Total Quality Management] explode all trees
#117 (health care or healthcare) NEXT/2 (quality or assurance or improvement)
#118 #113 or #114 or #115 or #116 or #117
#119 MeSH descriptor: [Benchmarking] explode all trees
#120 metric*
#121 Reference Standards
#122 quality indicator*
#123 (Outcome and process assessment)
#124 measurement*
#125 MeSH descriptor: [Health Services Research] explode all trees
#126 MeSH descriptor: [Quality Indicators, Health Care] explode all trees
#127 MeSH descriptor: [Health Status Indicators] explode all trees
#128 #119 or #120 or #121 or #122 or #123 or #124 or #125 or #126 or #127
#129 #118 and #128
#130 maternal health services
#131 Reproductive Health services
#132 child health services
#133 tuberculosis or HIV
#134 MeSH descriptor: [Communicable Diseases] explode all trees
#135 MeSH descriptor: [Chronic Disease] explode all trees
#136 MeSH descriptor: [Noncommunicable Diseases] explode all trees
#137 MeSH descriptor: [Mental Health Services] explode all trees
#138 hospital care
#139 surgical care
#140 MeSH descriptor: [Primary Health Care] explode all trees
#141 MeSH descriptor: [Nursing Care] explode all trees
#142 #130 or #131 or #132 or #133 or #134 or #135 or #136 or #137 or #138 or #139 or #140 or #141
#143 #142 and #129

2. Sup table

Table 3: MNCH, Mental Health and Primary Care studies use of methods to develop QoC metrics.

Studies which developed QoC metrics		Response	MNCH n = 24		Mental Health n=20		Primary Care n=47	
			No.	%	No.	%	No.	%
**Clear rationale for need for metrics**		Yes	22	90,9	20	100,0	46	97,9
		Not reported	2	9,1	0	0,0	1	2,1
**Approaches to developing QoC metrics**	**Literature or clinical guideline reviews**	Total reviews	20	83,3	18	90,0	32	68,1
		Not reported	4	16,7	2	10,0	15	31,9
		Systematic	3	12,5	6	30,0	8	17,0
	**Main methods to ID indicators**	Combined	11	45,8	18	90,0	16	34,0
		Reviews	11	45,8	2	10,0	23	48,9
		Existing metrics	0	0,0	0	0,0	6	12,8
		Routine data	2	8,3	0	0,0	2	4,3
	**Stakeholder Consultation**	Experts	9	37,5	13	65,0	19	40,4
		Providers	4	16,7	2	10,0	12	25,5
		Multiple stakeholders	9	37,5	3	15,0	8	17,0
		Not reported	3	12,5	2	10,0	8	17,0
	**Consultation methods used**	Rand/UCLA	8	33,3	4	20,0	12	25,5
		Delphi	5	20,8	7	35,0	10	21,3
		Nominal grp consensus	0	0,0	1	5,0	5	10,6
		Workshops	1	4,2	0	0,0	2	4,3
		Combination	4	16,7	3	15,0	5	10,6
		Other	6	25,0	5	25,0	3	6,4

		Not reported	-	-	-	-	10	21,3
	**Primary Study**	Total	19	79,2	19	95,0	21	42,6
		Surveys	6	25,0	11	55,0	10	21,3
		Descriptive	9	37,5	4	20,0	8	17,0
		Qualitative	4	16,7	3	15,0	3	6,4
		Not reported	5	20,8	2	10,0	26	55,3
	**Assessment of indicators**	Panel ratings	5	20,8	6	30,0	14	29,8
		Pilot or field testing	6	25,0	2	10,0	9	19,1
		Combinations of methods	7	29,2	7	35,0	7	14,9
		Other	3	12,5	2	10,0	4	8,5
		Not reported	3	12,5	3	15,0	13	27,7
**Clear criteria for selection of metrics**		Yes	20	83,3	17	85,0	40	85,1
		Not reported	2	8,3	2	10,0	5	10,6
**Testing of metrics done**	**Validity**	Yes	18	75,0	11	55,0	29	61,7
		N/A	2	8,3	2	10,0	3	6,4
	**Reliability**	Yes	15	62,5	6	30,0	19	40,4
		N/A	2	8,3	2	10,0	4	8,5
	**Feasibility**	Yes	18	75,0	13	65,0	25	53,2
		N/A	2	8,3	2	10,0	3	6,4
	**Usability**	Yes	14	58,3	3	15,0	14	29,8
		N/A	2	8,3	2	10,0	4	8,5
	**Related and competing measures reported**	Yes	5	20,8	0	0,0	5	10,6
		N/A	2	8,3	2	10,0	5	10,6

3. Definitions of selection criteria for quality of care indicators

- *Validity*: The indicator data elements are correct, and/or the indicator score correctly reflects the QoC provided, adequately identifying the differences in quality (NQF);
- *Reliability*: The indicator is well defined and precisely specified so that it can be implemented consistently within and across organisations and allows for comparability (NQF);
- *Feasibility*: The extent to which the specifications require data that are readily available or could be captured without undue burden and can be implemented for performance measurement (NQF);
- *Usability*: The extent to which potential audiences are using or could use performance results for both accountability and performance improvement (NQF);
- *Comparison to related and competing indicators*: If there are endorsed or new related indicators or competing indicators, the indicators are compared to address harmonization and/or selection of the best indicator (NQF);
- *Specification*: The indicator has defined components necessary to construct the indicator, including numerator, denominator and exclusions. The indicator has a minimum population level (NICE);
- *Acceptability*: The indicator assesses performance that is attributable to or within the control of the audience; and the results of the indicator can be used to improve practice (NICE);
- *Risk*: The indicator has an acceptable risk of unintended consequences (NICE).
